# Screening of deafness-causing DNA variants that are common in patients of European ancestry using a microarray-based approach

**DOI:** 10.1371/journal.pone.0169219

**Published:** 2017-03-08

**Authors:** Denise Yan, Guangxin Xiang, Xingping Chai, Jie Qing, Haiqiong Shang, Bing Zou, Rahul Mittal, Jun Shen, Richard J. H. Smith, Yao-Shan Fan, Susan H. Blanton, Mustafa Tekin, Cynthia Morton, Wanli Xing, Jing Cheng, Xue Zhong Liu

**Affiliations:** 1 Department of Otolaryngology, University of Miami Miller School of Medicine, Miami, Florida, United States of America; 2 National Engineering Research Center for Beijing Biochip Technology, Beijing, China; 3 Tsinghua University School of Medicine, Beijing, China; 4 Department of Pathology, Brigham and Women's Hospital and Harvard Medical School, Boston, Massachusetts, United States of America; 5 Laboratory for Molecular Medicine, Partners Personalized Medicine, Cambridge, Massachusetts, United States of America; 6 Department of Otolaryngology - Head and Neck Surgery, Carver College of Medicine, University of Iowa, Iowa City, Iowa, United States of America; 7 Department of Pathology, University of Miami Miller School of Medicine, Miami, Florida, United States of America; 8 Dr. John T. Macdonald Department of Human Genetics and John P.Hussman Institute for Human Genetics, University of Miami Miller School of Medicine, Miami, Florida, United States of America; 9 Department of Obstetrics, Gynecology and Reproductive Biology, Brigham and Women's Hospital and Harvard Medical School, Boston, Massachusetts, United States of America; 10 Division of Evolution and Genomic Science, School of Biological Sciences, Manchester Academic Health Science Center, University of Manchester, United Kingdom; NIDCR/NIH, UNITED STATES

## Abstract

The unparalleled heterogeneity in genetic causes of hearing loss along with remarkable differences in prevalence of causative variants among ethnic groups makes single gene tests technically inefficient. Although hundreds of genes have been reported to be associated with nonsyndromic hearing loss (NSHL), *GJB2*, *GJB6*, *SLC26A4*, and mitochondrial (mt) *MT-RNR1* and *MTTS* are the major contributors. In order to provide a faster, more comprehensive and cost effective assay, we constructed a DNA fluidic array, CapitalBioMiamiOtoArray, for the detection of sequence variants in five genes that are common in most populations of European descent. They consist of c.35delG, p.W44C, p.L90P, c.167delT (*GJB2*); 309kb deletion (*GJB6*); p.L236P, p.T416P (*SLC26A4*); and m.1555A>G, m.7444G>A (mtDNA). We have validated our hearing loss array by analyzing a total of 160 DNAs samples. Our results show 100% concordance between the fluidic array biochip-based approach and the established Sanger sequencing method, thus proving its robustness and reliability at a relatively low cost.

## Introduction

In industrialized nations, one child in 1,000 is born deaf, and an additional one in 300 births will be diagnosed with a milder degree of hearing loss. A further 1/1,000 children become profoundly hearing disabled before adulthood [1, 2]. Overall, hearing loss affects up to 8% of the world’s population [3, 4]. Hearing loss can be due to environmental or genetic factors, or a combination thereof. At least 50% of prelingual hearing loss is due to genetic factors in industrialized nations [5]. Non-syndromic hearing loss (NSHL) is the most common form of neurosensory deafness, and account for 70% of inherited hearing impairment. To date, more than 80 genes, with approximately 1,000 pathogenic variants, and 150 loci have been identified for NSHL (http://hereditaryhearingloss.org/). The spectrum of deafness-associated genetic variants varies greatly among regions and ethnicities [6, 7]. Mutations in *GJB2* are a major cause of autosomal recessive nonsyndromic hearing loss (ARNSHL) in many populations [8]. The most frequent pathogenic variants are c.35delG in European descent populations [9], c.167delT in Ashkenazi Jews [10], and c.235delC in East Asians (Japanese, Koreans, and Chinese) [11, 12, 13, 14]. In contrast, the *GJB6* deletion mutation del(GJB6-D13S1830) is common in France, Spain, the United Kingdom and Israel, accounting for 5.9–9.7% of all DFNB1 alleles [15]. Pathogenic variants in pendrin encoded by *SLC26A4* can cause both nonsyndromic (DFNB4) and syndromic deafness (Pendred syndrome; PDS). PDS is thought to be one of the most common forms of syndromic deafness and pathogenic variants of *SLC26A4* were reported to be the second most frequent cause of ARNSHL worldwide [16]. However, there are considerable differences in reported percentages of individuals segregating *SLC26A4* variants. The percentage of probands in whom biallelic variants are found varies accordingly, from a low of 13% [17] to a high of 62% [18]. This variation may be attributed to differences in selection criteria of each study and/or to the patient population being tested. Pathogenic variants in the mitochondrial (mt) 12S ribosomal RNA subunit gene (*MT-RNR1*) have been associated with amninoglycoside ototoxicity in an estimated 2% of deaf individuals in the United States [19, 20]. One of the most common mitochondrial variants is the m.1555A>G substitution in *MT-RNR1* which can be found in 0.6–2.5% of patients of European descent, 3–5% of Asians and as high as 17% of the Spanish population with NSHL [21]. The m.7444G>A mutations in the tRNA serine 1 gene (*MTTS1*) has been found in patients with maternally inherited sensorineural hearing loss, but is less likely to cause hypersensitivity to aminoglycoside.

The extraordinary genetic heterogeneity of hearing loss has been a great challenge for molecular testing: analysis of multiple genes using conventional gel-based and traditional Sanger sequencing techniques is expensive, time-consuming and cumbersome. The DNA microarray, or biochip, is a hybridization-based genotyping method that allows simultaneous multi-gene variant analysis.

Multiplexed microarray platforms provide parallel detection capabilities that make it ideally suited to genotyping of genetically heterogeneous conditions such as deafness. To achieve an efficient method for a genetic diagnosis of hearing loss, we developed a genetic hearing loss DNA chip, CapitalBioMiamiOtoArray, that allows simultaneous analysis of the nine most common mutations in patients of European descent in the genes *GJB2*, *GJB6*, *SLC26A4*, *MT-RNR1* and *MTTS1*. In this study we have validated our hearing loss biochip by analyzing a total of 160 DNA samples. Availability of an inexpensive and extensive screening test for common variants for hearing loss will result in affordable testing, improved diagnosis, more accurate genetic counseling, and eventually in improved management of hearing loss.

## Materials and methods

### Subjects

Genomic DNA was extracted from 136 whole blood samples and 24 dried blood spots. Of these, 101 were from deaf patients and 59 from normal hearing controls. For validation, 60 samples were pre-tested for the specific variants in *GJB2*, *GJB6* and *SLC26A4* genes by Sanger sequencing. Mitochondrial DNA (mtDNA) genes were subjected to PCR-RFLP (Restriction Fragment Length Polymorphism) analysis using specific primers [22]. The diagnosis of sensorineural hearing loss was established via standard audiometry in a sound-proofed room according to current clinical standards. HL was congenital or prelingual-onset with a severity ranging from moderate to profound. Clinical evaluation included a thorough physical examination and otoscopy in all cases. This study was approved by the University of Miami Institutional Review Board, USA (permit #20010415). The cohort of participants was recruited between 2003 and 2016. A signed informed-consent form was obtained from each subject or, in the case of a minor, from the parents.

### The deafness fluidic microarray

The hearing loss fluidic array, CapitalBioMiamiOtoArray (CapitalBio Corporation, Beijing, China) was designed to detect the following nucleotide changes: *GJB2* [c.35delG, p.W44C (c.132G>C), p.L90P (c.269T>C), c.167delT]; *GJB6* c.309kb deletion; *SLC26A4* [p.L236P (c.707T>C), p.T416P (c.1246 A>C)]; and mtDNA m.1555A>G and m.7444G>A mutations. The nine mutations were selected based on their high prevalence in patients of European descent. The 167delT is common in Ashkenazi Jews. The mtDNA changes 1555A>G and 7444G>A are frequently found in southern European populations [22, 23, 24, 25].

The Amplification refractory mutation system (ARMS) technique for detecting known point mutations described by Newton et al [26] was applied. The method requires that 3'-OH terminal mismatched primers are refractory to extension by the DNA polymerase and thus the terminal 3'-nucleotide only of a PCR primer be allele specific; the primer is synthesized in two forms. The wild type form is refractory to PCR on mutant template DNA and the 'mutant' form is refractory to PCR on normal DNA. We have previously described in details the design, optimization and validation of tagged primers and multiplex PCR conditions [27, 28, 29] using allele-specific amplification primers to the wild type (WT) and variant sequences (M) and common primers (S1 and S2 Tables).

**Fig 1:**
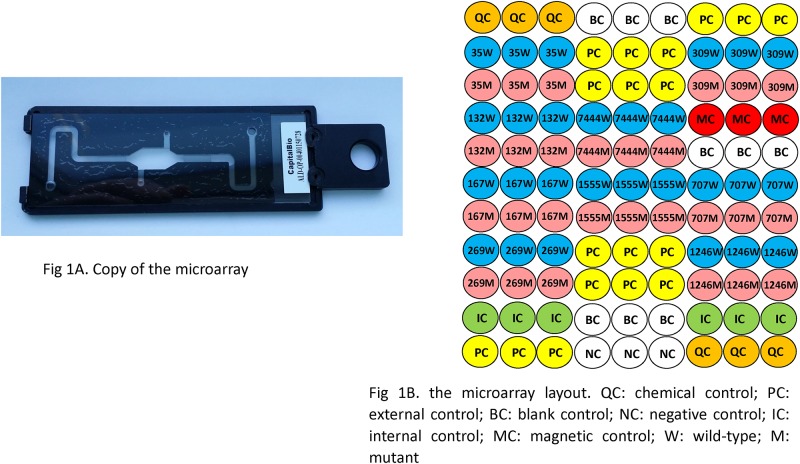
Microarray design. (A) Copy of the microarray; (B) the microarray layout. QC: chemical control; PC: external control; BC: blank control; NC: negative control; IC: internal control; MC: magnetic control; W: wild-type; M: mutant.

The microarray is composed of a fluidic manifold made of polycarbonate glued on the glass slide to introduce a dynamic hybridization for signal enhancement and acceleration of reaction kinetics (Fig 1A). The tag probes contain a poly (dT) spacer and a 5′ amino group, are diluted in 50% dimethyl sulfoxide (DMSO) to a final concentration of 15 micromole and are spotted with a SmartArrayer 136 spotter (CapitalBio) on the aldehyde-functionalized glass slides. Each tag probe is spotted in triplicate. The diameter of each spot is approximately 150 micrometers. The pattern of the distribution of probes on the microarray is shown in the microarray layout (Fig 1B) (Fig 1A and 1B: Microarray design); the different numerals refer to different genetic loci, whereas upper case alphabetic letters W and M represent wild-type and mutant-type variant probes, respectively. A number of control probes are also represented: QC and NC are the positive and negative controls for the spotting of probes during array manufacture, PC and BC are the positive and negative controls for the hybridization reaction, IC is the internal control for the gene amplification, and MC is the positive control for magnetic beads. The QC, IC, BC and PC were spotted in 6 to 12 replicates.

#### Magnetic bead-based DNA microarray analysis

**Fig 2:**
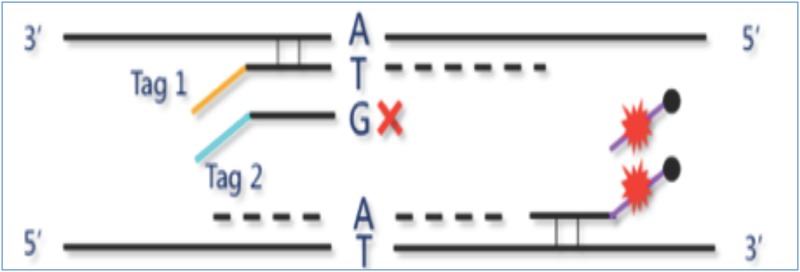
The principle for design of the amplification primers. Multiplex PCR is performed as described previously [27, 28, 29]. Asymmetric PCR procedure was used for generation of labeled single-stranded DNA in excess for hybridization and detection.

Mutations were detected using the nine common deafness mutations detection kit for populations of European descent (CapitalBio, Beijing). Each DNA sample was amplified in two 25-μl multiplex PCR reactions (A and B) as recommended by the manufacturer. Amplification primers were designed according to the principle shown in Fig 2 (Fig 2. The principle for design of the amplification primers). Each PCR reaction is prepared by combining 5 μl template DNA (15–150 ng), 10 μl mixture of amplification primer and 10 μl mixture of amplification reagent to the 25 μl reaction system individually. For degradation of carryover contaminating molecules prior to amplification, we applied a procedure that makes use of dUTP incorporation instead of dTTP during amplification, which is preceded by treatment of PCR reaction mixtures with uracil-N-glycosylase (UNG) at 37°C to degrade uridine-containing carryover products [30], and subsequent cleavage of apyrimidinic polynucleotides at elevated temperature (95°C for 15 min) to remove contaminating U-DNA from the sample. The amplification was performed in a Master Cycler nexus X2e (Oppendorf) by setting the temperature rate using the RAMP function of the PCR instrument under the following conditions: 37°C for 10min, 95°C for 15 min; 96°C for 1 min; then: 94°C for 30 sec, ramp (0.4 C/s) to 55°C, hold for 30 sec; ramp (0.2 C/s) to 70°C, hold for 45 sec for 32 cycles; followed by 60°C for 10 min, 4°C soak. The total reaction time was about 3 hours and 20 minutes. Amplified products from the two tubes were mixed, and the amplicons were then captured by magnetic beads. Captured amplicons were denatured by NaOH, and only the single stranded DNA attached to the magnetic beads was collected and dissolved in hybridization buffer. Approximately 30 μL of the hybridization solution was then injected into the sample inlet to cover the central hexagonal array region. The sample inlet and the air outlet were then sealed and the chip was placed into the Easy Array 3A (CapitalBio Corporation) for a dynamic hybridization at 55°C for 20 min followed by washing at 42°C for 2 min. Due to the flowing reaction controlled by the instrument, the hybridization and washing process was about four times faster than the conventional static diffusion reaction. The total time required for PCR amplification, hybridization, washing and detection was about 4 hours.

## Results

DNA from 101 deaf patients was used to validate the deafness chip. Of these, 60 subjects were pre-screened and known to be positive for one or two specific variants (s) present on the chip; 18 had one *GJB2* variant, 21 had two *GJB2* variants (homozygous or compound heterozygous), four were digenic for *GJB2*-c.35delG and *GJB6*-309kb deletion. For *SLC26A4*, 10 of the subjects were heterozygous and two were homozygous for variants on the chip. All the five individuals tested positive for the m.1555A>G variant in *MT-RNR1* are homoplasmic as well as two deaf subjects among the 41 cases of unknown molecular genetic etiology (Table 1).

**Table 1:** Mutations detected in the deaf individuals.

Gene	Genotype	Number of Subjects
*GJB2*	35delG/35delG	11
*GJB2*	35delG/WT	10
*GJB2*	167delT/167delT	2
*GJB2*	167delT/WT	2
*GJB2*	L90P/L90P	5
*GJB2*	L90P/WT	6
*GJB2*	35delG/L90P	3
*SLC26A4*	L236P/WT	5
*SLC26A4*	T416P/T416P	2
*SLC26A4*	T416P/WT	1
*SLC26A4*	L236P/(c.1001+1 G>A)	2
*SLC26A4*	T416P/(c.412G>T)	2
*MTRNR1*	1555A>G/1555A>G	7
*GJB2/GJB6*	35delG/309kb	4
		62

The results showed 100% concordance between the fluidic array biochip-based approach and the established PCR protocol. It did not detected any unreported variant in the validation set as expected Of 10 individuals found with one *SLC26A4* variants, two were biallelic based on Sanger Sequencing, because the c.1001+1 G>A and c.412G>T variants in these two individuals were not present on the chip (Table 1). There were no false-positive results in 59 negative controls.

## Discussion

The profound genetic heterogeneity of deafness has proved a challenge for genetic testing using conventional approaches. It is neither financially or nor practically feasible to screen for causative variants in many cases, because they can be in one of several hundred genes, many of which have many exons. Demand for genetic testing for deafness has risen dramatically over the last decade, in part due to identification of many genes associated with hearing loss and to the introduction of Universal Newborn Hearing Screening Programs in many countries. There is therefore a need for simpler, cheaper, and more comprehensive methods for screening for variants associated with hearing loss.

Patterns of high frequency pathogenic variants in diverse populations and their population-wide distributions make it efficacious to target ethnically restricted disease variants in screening tests. In the present study, we have used an oligonucleotide-array based chip approach for simultaneous analysis of nine mutations in five genes. Using this chip, rapid screening for specific variants at different genetic loci can be performed. The technology can also easily be expanded or modified, according to further epidemiologic surveys of carrier rate and the spectrum of genetic variants associated with hearing loss in certain ethnic backgrounds. We have validated the hearing loss chip on DNA obtained from 60 deaf individuals who were previously screened and known to carry one or two pathogenic variants included on the chip. Analysis detected all known variants in the correct zygotic and plasmic state in nuclear and mitochondrial genes. These results prove the accuracy and reliability of the custom capture experiment.

Population-based genetic testing has been proposed as a major vehicle for translating genetic and genomic advances into human health and disease. Testing for pathogenic variants in common deafness causing genes has several potential advantages over conventional hearing loss evaluation without genetic testing. Anticipated benefits from genetic screening include: 1) A need for more expensive or invasive procedures may be precluded, such as tests to identify prenatal infections, electrocardiograms and other heart and thyroid function tests. Inner ear imaging techniques such as magnetic resonance imaging (MRI) or a computed tomography (CT) scan can be avoided; 2) Earlier detection of hearing loss for making informed choices of interventions such as hearing aids, cochlear implants, or sign language proven to be effective in significantly improving language ability and quality of life outcomes can be realized. Research evidence has demonstrated the practicability, cost-efficiency and benefits of universal newborn hearing screening (UNHS) [1, 9, 31, 32, 33]. However, UNHS programs may suffer from inherent limitations such as failure to detect children with slight or mild hearing loss, because the target condition for the majority of UNHS programs is permanent hearing loss >35dB [34]. Additionally, children with late-onset or progressive hearing loss may be missed by UNHS, because their hearing is normal or near-normal at birth. Lifetime costs of all care related to deafness and lost productivity have been estimated to be $1.1 million USD per birth cohort of 80,000 children [35]; 3) Predictive information about possible progression of a hearing disorder may be provided, which can facilitate parents and professional teams in implementation of an individualized health-maintenance strategy; 4) Monitoring of associated clinical manifestations and complications, particularly for certain syndromic forms of hearing loss for early management may be enabled; 5) Obtaining an etiologic diagnosis provides the basis for precise genetic counseling including information on the chance of recurrence in the family that can inform reproductive decisions. Lastly, 6) Information pertinent to aminoglycoside antibiotic ototoxicity risk factors can guide medical care (e.g., avoiding administration of aminoglycoside antibiotics among those with the MTRNR1 m.1555A>G variant to prevent hearing loss.

Continuous advances in DNA sequencing technologies have allowed interrogation of very large numbers of variants in a highly rapid and inexpensive manner. As knowledge of the variant spectrum in specific populations increases, selection of variants in a population specific manner can be made. Routine screening can thus be performed at a reduced cost for a larger number of samples.

There are many reliable, relatively simple and inexpensive variant detection techniques available for a faster, more comprehensive and cost-effective approach for NSNHL mutations. These include the HHL APEX (Hereditary Hearing Loss Arrayed Primer Extension) microarray [36], that is comprised of 198 variants in six nuclear genes *(GJB2*, *GJB6*, *GJB3*, *GJA1*, *SLC26A4* and *SLC26A5*) and two mitochondrial genes (*MTRNR1* and *MTTS1*). Considering that the APEX array did not detect a single *GJB3* or *GJA1* sequence variant in any of the study subjects, these gap junction proteins do not seem to be a major cause of NSHL in populations of European descent. Inclusion of *GJB3* for pre-screening is thus not warranted. For *SLC26A5*, the IVS2-2A>G (c.-53-2A>G) variant was found only in a heterozygous state, however, its clinical significance is unknown and it has been found at the same frequency in deaf individuals and in a control group and pathogenicity seems therefore now questionable [37, 38]. Resequencing microarrays have also been developed by Affymetrix [39, 40], allowing for both detection of previously reported hearing loss variants and discovery of new variants. But the Affymetrix resequencing array for variants screening is costly in comparison with the other methods, limiting its implementation in the routine laboratory setting [40, 41]. In addition, a key drawback of this technology has been a high false positive rate (~70%) among identified variants, thus requiring time-consuming and expensive [39]. The TaqMan OpenArray has also been described [42]. All of these technologies have in common the use of oligonucleotides probes applied on a solid support and fluorescent compounds. The APEX microarray as well as our fluidic array allow some versatility, because it can be easily modified to alter mutation composition and/or to increase the number of mutations. In contrast, for OpenArray and resequencing arrays, modifications cannot be made after manufacturingof the plates/chips.

In this present study we have used an allele-specific PCR-based universal array designed to simultaneously screen nine deafness associated variants in the *GJB2*, *GJB6*, *SLC26A4*, *MT-RNR1* and *MTTS* genes that are common in patients of European descent. The test results are determined based on the fluorescent hybridization signal and the distribution of microarray probe. The array results for the 60 pre-screened subjects were consistent with the previous molecular diagnostic testing data and there were no false-positive results in 59 negative controls, thus confirming the100% sensitivity and reliability of the assay.

A direct comparison of cost-effectiveness of Miami-CapitalBio array mutation analysis with other array-based tests is not straightforward since the number of genes and mutations covered vary depending on platform used. Furthermore, laboratory practices and workflows, array designs, and analytical performance parameters, all of which impact test cost, can differ greatly. For genetically heterogeneous diseases such as hearing loss, testing laboratories must strive to increase clinical sensitivity by including more genes and minimizing test price and turn around time. An Affymatrix resequencing microarray capable of resequencing 13 deafness causative genes has been reported to allow an individual worker to produce a throughput of nearly 100 patients per month and has reduced the cost of testing of the 13 genes roughly in half compared to dideoxy capillary sequencing approaches [39, 43].

Use of the Miami-CapitalBio fluidic array is a good option as an initial screening tool for the most common variants within genes. The cost of routine research grade Sanger sequencing of each variant in *GJB6*, *SLC26A4 and MT-RNR1* is approximately US$5 per sample. The cost estimated for analysis of all the 9 variants per patient, including materials and reagents, is approximately US$ 30. Costs for technician labor were not calculated directly since salaries, benefits, and overhead rates vary across laboratories, but we estimated that a single technician could process 50 samples per week including data analysis. Equipment and maintenance costs are expected to be minimal as the array protocol only requires equipment that is of current standard for molecular laboratories. Overall, we demonstrate that the use of CapitalBioMiamiOtoArray for analysis of common mutations in patients of European descent is cost effective and the genotypes produced are high quality and match those collected using the established Sanger sequencing at a rate of 100%.

## Supporting information

S1 Table Loci and allele-specific amplification primers to the wild type (WT) and variant (MT) sequences. Tag sequences are underlined and mismatched bases are highlighted.
(XLSX)

S2 Table Common primers used for amplification. F: forward; R: reverse; BT: bitotin.
(XLSX)
